# Correction: Enhancing osteogenic differentiation of MC3T3-E1 cells during inflammation using UPPE/β-TCP/TTC composites *via* the Wnt/β-catenin pathway

**DOI:** 10.1039/d4ra90004a

**Published:** 2024-01-24

**Authors:** Qi-lin Li, Ya-xin Wu, Yu-xiao Zhang, Jing Mao, Zhi-xing Zhang

**Affiliations:** a Department of Stomatology, Tongji Hospital, Tongji Medical College, Huazhong University of Science and Technology Wuhan 430030 China maojing@hust.edu.cn zzx@tjh.tjmu.edu.cn; b School of Stomatology, Tongji Medical College, Huazhong University of Science and Technology Wuhan 430030 China; c Hubei Province Key Laboratory of Oral and Maxillofacial Development and Regeneration Wuhan 430022 China

## Abstract

Correction for ‘Enhancing osteogenic differentiation of MC3T3-E1 cells during inflammation using UPPE/β-TCP/TTC composites *via* the Wnt/β-catenin pathway’ by Qi-lin Li *et al.*, *RSC Adv.*, 2024, **14**, 1527–1537, https://doi.org/10.1039/D3RA05529A.

The authors regret that an incorrect version of Fig. 4 was included in the original article. The correct version of Fig. 4 is presented below.

**Fig. 4:**
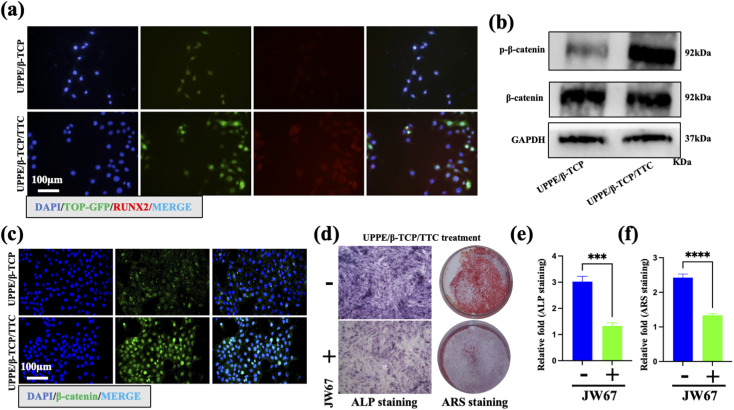
UPPE/β-TCP/TTC composites enhanced the osteogenic differentiation of MC3T3-E1 cells through the Wnt/β-catenin pathway under *P.g*-LPS stimulation. (a) Immunofluorescent staining of TOP-GFP and RUNX2 in MC3T3-E1 cells on the surface of UPPE/β-TCP and UPPE/β-TCP + 1% TTC after cell culture in *P.g*-LPS for 21 days. (b) The protein expression levels of β-catenin and p-β-catenin on the surface of UPPE/β-TCP and UPPE/β-TCP + 1% TTC after cell culture in *P.g*-LPS for 21 days. (c) Immunofluorescent staining of β-catenin in MC3T3-E1 cells on the surface of UPPE/β-TCP and UPPE/β-TCP + 1% TTC after cell culture in *P.g*-LPS for 21 days. (d) ARS and ALP staining of MC3T3-E1 cells (with or without JW67, the WNT pathway inhibitors) on the surface of UPPE/β-TCP + 1% TTC after cell culture in *P.g*-LPS for 21 days and quantitative analysis in (e) and (f). ***P < 0.001, ****P < 0.0001, compared with the without JW67 group.

The Royal Society of Chemistry apologises for these errors and any consequent inconvenience to authors and readers.

## Supplementary Material

